# Circ_ZNF124 promotes non-small cell lung cancer progression by abolishing miR-337-3p mediated downregulation of JAK2/STAT3 signaling pathway

**DOI:** 10.1186/s12935-019-1011-y

**Published:** 2019-11-14

**Authors:** Qianping Li, Qin Huang, Shaofei Cheng, Song Wu, Hongyang Sang, Junyi Hou

**Affiliations:** 1 Department of Cardiothoracic Surgery, Shanghai Sixth People’s Hospital East Affiliated to Shanghai University of Medicine & Health Sciences, 222 Huanhu Xi San Road, Pudong New District, Shanghai, China; 2 Department of Pathology, Shanghai Sixth People’s Hospital East Affiliated to Shanghai University of Medicine & Health Sciences, 222 Huanhu Xi San Road, Pudong New District, Shanghai, China; 3 Department of Gastroenterology, Shanghai Sixth People’s Hospital East Affiliated to Shanghai University of Medicine & Health Sciences, 222 Huanhu Xi San Road, Pudong New District, Shanghai, Zip:201306 China

**Keywords:** NSCLC, Circ_ZNF124, miR-337-3p, JAK2/STAT3

## Abstract

**Background:**

Previous genome-wide transcriptome profiling found circ_ZNF124 was highly expressed in lung adenocarcinoma, however, the role of circ_ZNF124 in non-small cell lung cancer (NSCLC) is still unknown. The purpose of this study was to investigate the role and molecular mechanism of circ_ZNF124 in NSCLC development.

**Methods:**

The expression of circ_ZNF124, miR-337-3p and JAK2 (Janus Kinase 2) in lung cancer cell lines and normal epithelial cells were detected by qRT-PCR (quantitative real-time PCR). siRNA was used to knockdown circ_ZNF124 expression in cells. The effects of circ_ZNF124 in NSCLC cells were determined by cell growth, cell migration, cell cycle analysis and colony formation. Bioinformatics analysis, RNA immunoprecipitation, luciferase assay and western blots were used to study the molecular mechanism of circ_ZNF124 in NSCLC.

**Results:**

The results showed that circ_ZNF124 expression was highly upregulated in NSCLC cells than in normal epithelial cells. Knockdown of circ_ZNF124 by using siRNA significantly decreased cell growth, promoted cell cycle arrested in sub-G1 phase, impaired cell migration and colony formation. Bioinformatic analysis discovered that miR-337-3p was a direct target of circ_ZNF124. In contrast to circ_ZNF124, miR-337-3p expression was significantly downregulated in NSCLC cells. Biotin labeled circ_ZNF124 immunoprecipitation and luciferase assay showed that miR-337-3p could directly bind to and affect circ_ZNF124 activity. The regulation of circ_ZNF124 on miR-337-3p was also investigated. Further analysis showed that despite STAT3 (signal transducer and activator of transcription 3), JAK2 was also a target of miR-337-3p, overexpression of miR-337-3p greatly downregulated JAK2, STAT3 and JAK2/STAT3 downstream regulated oncogenes HIF1a (Hypoxia-inducible factor 1-alpha), BCL2 (B cell lymphoma 2) and c-FOS expression, however, the roles of miR-337-3p in JAK2/STAT3 signaling pathway were greatly inhibited in the presence of circ_ZNF124.

**Conclusion:**

In NSCLC, highly expressed circ_ZNF124 promoted the activation of JAK2/STAT3 signaling pathway by acting as a sponge of miR-337-3p, thus promoting the occurrence and development of NSCLC. Circ_ZNF124 could be a potential biomarker or target for the treatment of NSCLC patients in the future.

## Background

Lung cancer is one of the most common malignant types of tumor. Annually, over one million deaths were associated with lung cancer worldwide [7]. Since early stages of lung cancer does not cause any signs and symptoms, the diagnosis is usually made at advanced stages of the disease, thus, the overall 5 year survival rate is pretty low with only around 20% [3, 30]. NSCLC is a subtype of lung cancer with a poor prognosis [12]. Approximately, 85% of lung cancer cases are NSCLC [2, 27]. Due to the lack of sensitive early diagnosis makers and molecular targets, treatment of NSCLC patients is still not efficient. Exploring the underlying pathogenesis molecular mechanism and identifying novel diagnostic biomarkers are urgent for NSCLC treatment.

CircRNA is a group of circular RNAs derived from pre-mRNAs [6]. In structure, they are characterized by single strand, covalently-closed loop RNAs with no Poly A tail. Due to the circular structure they are highly stable in vivo. The generation of circRNAs can be achieved through ‘exon skipping’ or ‘direct back-splicing’ [16, 17, 32]. The primary role of circRNAs discovered so far is acting as miRNAs sponge. For example, a well-studied circular RNA CDR1as was found to have 73 seed binding sites for miR-7 [14]. Knockdown of CDR1as in mouse decreased miR-7 and upregulated miR-7 targeted genes expression [31]. However, despite few well studied circular RNAs, the functions of most circular RNAs remain largely unknown.

Recently more and more studies suggested that circRNAs might play important roles in tumor development [18]. Studies on NSCLC have shown that some dysregulated circRNAs were tightly associated with NSCLC occurrence and metastasis. For example, circular RNA ATXN7 has been reported to promote NSCLC cell lines growth, metastasis, and high circATXN7 expression in NSCLC patients were associated with poorer survival rates compared with lower circATXN7 [15]. Circular RNA microarray on 52 NSCLC patients treated with gefitinib found that 1377 circRNAs were differentially expressed in gefitinib effective and ineffective groups, further study showed that elevated hsa_circ_0109320 was associated with longer progression-free survival in gefitinib-treated NSCLC patients, indicating that hsa_circ_0109320 might be a potential biomarker for prognosis in gefitinib treated NSCLC patients [24].

Previous genome wide ribosomal RNA‐depleted RNA sequencing with paired lung adenocarcinoma and non-tumor tissues found that over 50 circular RNAs were differentially expressed [11]. Among them, cir_ZNF124 was found to be highly expressed in lung adenocarcinoma. However, the expression and role of circ_ZNF124 in NSCLC are still unclear. In this study we confirmed that circ_ZNF124 was highly expressed in NSCLC cells compared with normal cell, and high circ_ZNF124 expression promoted NSCLC cells proliferation. Further mechanism studies showed that the role of circ_ZNF124 in facilitating NSCLC progression was through inhibiting miR-337-3p and activating JAK2/STAT3 signaling pathway.

## Materials and methods

### Cell lines

Lung cancer cell lines H1975, H1299, HCC827, A549 and normal immortalized epithelial cell type BEAS-2B were used for in vitro study. H1975, H1299, HCC827 were maintained in Roswell Park Memorial Institute (RPMI-1640) medium (Gibco) added with 10% fetal bovine serum (FBS), A549 (F-12) was maintained in F-12K medium (ATCC) containing 10% FBS, BEAS-2B was cultured in Bronchial Epithelial Cell Growth Medium (Lonza). All the cells were maintained at 37 °C with 5% CO_2_.

### Reporter assay

The wild-type circ_ZNF124 or 3′–untrans-lated region (3′–UTR) of JAK2 containing the miR-337-3p binding site was cloned into the pGL3–control vector. circ_ZNF124 and JAK2-3′UTR mutants with miR-337-3p binding sites mutation were generated by using Q5 Site-Directed Mutagenesis kit (NEB, USA). All plasmids were confirmed by sequencing. Lipofectamine 2000 (Invitrogen; Thermo Fisher Scientific, Inc.) was used to transfect plasmids into cells. In brief, 0.5 million cells were seeded into 6 well plates 1 day before transfection. 50 ng luciferase vector, 5 ng Renilla vector and different amounts of miR-337-3p mimic or circ_ZNF124 overexpression vector as indicated were transfected into cells with lipofectamine 2000 according to the manufacturer’s instructions. After 36 h, cells were harvested and washed with PBS (Phosphate-buffered saline) once. Luciferase activity was determined by using a Dual-Luciferase reporter assay kit (Promega Corporation, USA). The values of Renilla were used to normalize luciferase activity.

### Oligonucleotide transfection

miR-337-3p mimic and negative control were purchased from ThermoFisher, siRNA targeting circ_ZNF114 was synthesized by Ribobio (Guangzhou, China). Cell transfection was performed with Lipofectamine RNAiMax (Life Technologies) according to manufacturer’s instructions.

### Western blot

Cells were harvested and washed once with PBS. Protein was extracted by using RIPA (Radioimmunoprecipitation assay buffer) lysis buffer (10 mM Tris, pH 7.4. 100 mM NaCl. 1 mM EDTA. 1 mM EGTA. 1% Triton X-100. 10% glycerol. 0.1% SDS. 0.5% deoxycholate.) containing protein inhibitors followed by ultracentrifuge at 13,000 rpm for 5 min. Supernatants were collected, and protein was quantitated by BSA assay. Proteins with different molecular weight were then separated by SDS-PAGE (sodium dodecyl sulphate-polyacrylamide gel electrophoresis) electrophoresis and transferred to NC (Nitroellulose) membrane. Membrane was blotted with 5% non-fat milk. Primary antibodies targeting proteins of interests were incubated with membrane with shaking at 4 °C overnight. The next day, primary antibodies were collected, and membrane was continue incubated with HRP tagged secondary antibodies at room temperature (RT) for 1 h. Membrane was imaged with Odyssey CLx.

### RNA immunoprecipitation (RIP)

RNA immunoprecipitation used to verify circ_ZNF124 and miR-337-3p interaction in vivo was performed by using Magna RIP RNA-Binding Protein Immunoprecipitation Kit (Millipore, Bedford, MA). In brief, 20 million A549 cells were harvested and lysed with RIP lysis buffer containing protease and RNase inhibitors. Cell lysates were then split, and RNA was pulled down by incubating with anti-Argonaute 2 (AGO2) antibody (Millipore) or control rabbit IgG (Millipore), followed by rotating at 4 °C overnight. After proteinase K treatment, the immunoprecipitated RNAs were extracted. The abundance of circ_ZNF124 and miR-337-3p were determined by qRT-PCR.

### Cell growth and cell cycle

After transfection for 24 h, cells were harvested and seeded into 10 cm dish at 0.1 million cells/ml. At days 1, 2, 3 and 5 cells were collected and counted with Hemacytometers. At least 3 replicates were performed. CellTiter-Glo (CTG) luminescent cell viability assay used to measure cell viability was performed at day 5 after cell outgrowth. In brief, cells were trypsin digested and washed once with PBS. Cells was re-suspended with 500 µl PBS. 30 µl cell suspension was aspirated and transferred into 96 well white plate, CTG reagent was added to cells followed by incubation at RT for 10 min, cell viability was recorded with Luminometer.

Cell cycle was measured with propidium iodide (PI) staining. In brief, harvested cells were washed with PBS and re-suspended with 100 µl cold PBS. Cells were fixed with 900 µl cold ethanol by adding dropwise with vortex. Cell fixation was performed at 4 °C for at least 2 h. Fixed cells were stained with 500 µl PI staining solution (50 µg/ml; 1 mg/ml of RNase A, 0.1% Triton X-100 in PBS) at RT for 30 min. FACS was then applied to detect cell cycle.

### Cell colony formation

A549 and H1299 cells were transfected with Circ_ZNF124 siRNA or control siRNA. After 24 h, cells were collected and counted. Cells were seeded into 6-well plates and continue culture in complete medium at 37 °C with 5% CO_2_ for 2 weeks until the cell colonies were clearly observed. Colonies were first fixed with methanol for 10 min followed by staining with crystal violet (0.1% concentration; comWin Biotech, Beijing) for 5 min at RT. Stained colonies were recorded with scanner and colony numbers were counted for statistical analysis.

### Cell migration

After transfection for 24 h, cells were harvested and seeded into 6 well plates. The next day, sterile 200 µl sharp tips were used to draw lines on the cell. Cell migration was determined 24 h after cell scratch. At least 10 views per well were recorded for statistical analysis.

### RNA extraction and qRT-PCR

Total RNA was extracted by using TRIzol (Thermo Fisher, USA). 1 µg RNA was used to reverse transcript to cDNA by using PrimeScript RT Master Mix (Takara, Japan). The expression of circ_ZNF124, JAK2, HIF1a, BCL2, c-FOS and miR-337-3p were determined by real-time PCR analyses by using SYBR master mix (Bio-rad). U6 small nuclear RNA levels served as an internal control for miRNA expression detection. GAPDH (Glyceraldehyde 3-phosphate dehydrogenase) was used as an internal control for gene expression. The relative RNA expression level was calculated with 2^−∆∆ct^. Primers used were listed in Table 1.

**Table 1:** The sequences of specific primers

Names	Sequences (5′–3′)
GAPDH-F	GTAGAGCGGCCGCCATG
GAPDH-R	GATTTCCATTGATGACAAGC
qRT-GAPDH-F	ACTTCAACAGCGACACCCACTC
qRT-GAPDH-R	TCTCTTCCTCTTGTGCTCTTGCT
qRT-HIF1A-F	GAACGTCGAAAAGAAAAGTCTCG
qRT-HIF1A-R	CCTTATCAAGATGCGAACTCACA
qRT-cFOS-F	CCGGGGATAGCCTCTCTTACT
qRT-cFOS-R	CCAGGTCCGTGCAGAAGTC
qRT-BCL2-F	GGTGGGGTCATGTGTGTGG
qRT-BCL2-R	CGGTTCAGGTACTCAGTCATCC
qRT-circZNF_124-F	GACCAGAGCATTGAAGA
qRT-circZNF_124-R	AGGATCCAACAAAGCC
qRT-JAK2-F	ATCCACCCAACCATGTCTTCC
qRT-JAK2-R	ATTCCATGCCGATAGGCTCTG
JAK2sg1-F	CACCGAGAAAACGATCAAACCCCAC
JAK2sg1-R	AAACGTGGGGTTTGATCGTTTTCTC
JAK2-3′UTR-F	AACAGATCTGTTTTCTAAT TTTTCC
JAK2-3′UTR-R	GTTAGATCTCAACAACG AACAACCCCT
circ_ZNF124-pGL3-F	AGACGTGATGCAGG
circ_ZNF124-pGL3-R	GGATCCAACAAAGCC
qRT-U6-F	TTGGTCTGATCTGGCACATATAC
qRT-U6-R	AAAAATATGGAGCGCTTCACG
qRT-miR-377-3p-F	CGCAGCTTCTTTCCGTAGT
qRT-miR-377-3p-R	GGTCCAGTTTTTTTTTTTTTTTGAG
JAK2-3′UTR-mirmutant-F	GTTTTATTTGGTCGACAAATTTCCCTGACCCTAAATAATAC
JAK2-3′UTR-mirmutant-R	TAGACATTTGTTCCCTTTATG
circ_ZNF124-mirmutant-F	TCTGGCTTCCGACAAAAACAAAGGGGAAGAC
circ_ZNF124-mirmutant-R	TTCCTGAAGGTTTCCTGC

### Biotin pull down

Wild type circ_ZNF124 probe and miR-337-3p binding site mutated circ_ZNF124 probe were labeled with biotin and incubate with Streptavidin magnetic beads to generate probe-coated beads. 10 million A549 cells were lysed and incubated with probe-coated beads with shaking at 4 °C overnight. RNA bound to the beads was eluted. The enrichment of miR-337-3p was evaluated by qRT-PCR.

### CRISPR (clustered regularly interspaced short palindromic repeats)

Lentiviruses expressing Cas9 were made by transfecting 293T cells with VSVG, PsPAX2 and lentiCas9-Blast. Lentiviruses were harvested at day 1 and day 2 after transfection, harvested viruses were used to infect A549 and H1975. Cells stably expressing Cas9 were selected by blasticidin for 5 days. The expression of Cas9 was verified by western blot. To delete JAK2, sgRNA targeting JAK2 was cloned into pLentiGuide-puro vector and packaged into lentiviruses with VSVG, PsPAX2 in 293T cells. Harvested lentiviruses were then used to infect A549 and H1945 cells in which Cas9 were stably expressed. After transfection, puromycin was used to select lentiviruses infected cells. The efficiency of JAK2 deletion was tested by western blot after puromycin selection for 3 days.

### Bioinformatics

Circinteractome (https://circinteractome.nia.nih.gov/) was used to predict the possible circ_ZNF124-miRNA interactions. miRDB and TargetScan (http://www.targetscan.org/mamm_31/) were used to search potential miRNA targeting genes.

### Statistical analysis

Statistical analysis was performed using SPSS 20.0 software (Chicago, IL, USA). Student’s t test was applied to compare the differences between experimental group and control group. All data were shown as mean ± SEM. *P < 0.05 was considered statistically significant.

## Results

### Circ_ZNF124 is highly expressed in NSCLC cell lines

**Fig. 1:**
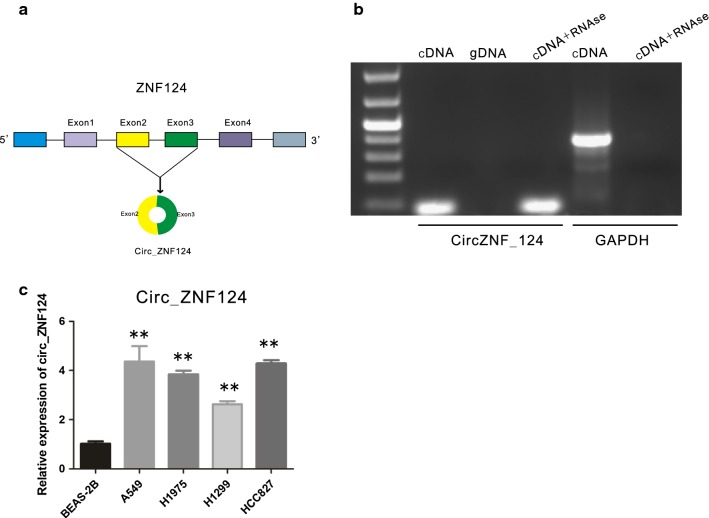
Circ_ZNF124 expression was up-regulated in NSCLC cells. **a** Diagram showed that circ_ZNF124 was back-spliced by ZNF124 exons 2 and 3. **b** PCR results of circ_ZNF124 amplification before and after RNase treatment from gDNA or cDNA by divergent primers. GAPDH was used as a negative control. **c** qRT-PCR results of circ_ZNF124 expression. *NSCLC* non-small cell lung cancer. *P < 0.05, **P < 0.01

Previous genome wide circular RNA sequencing identified many differentially expressed circular RNAs between lung adenocarcinoma tumor tissues and normal tissues [11]. In particular, hsa_circ_0017348 derived from ZNF124 was found highly overexpressed in lung adenocarcinoma tumor tissues. Further sequence analysis found this circular RNA was back spliced by exon 2 and 3 of ZNF124 transcripts (Fig. 1a). To confirm the existence of circ_ZNF124 in NSCLC, divergent primers were used to amplify circularized ZNF124. RNase A was used to digest liner RNA while keep circular RNA intact. As indicated, divergent primers can amplify desired length of DNA fragment from cDNA but not from gDNA (genomic DNA). Furthermore, circ_ZNF124 could still be amplified when treated RNA with RNase A before reverse transcription, while GAPDH could not be detected after RNase A treatment, indicating that circ_ZNF124 was stably exist in NSCLC cells (Fig. 1b). Sanger sequencing confirmed these PCR products.

To investigate the expression of circ_ZNF124 in NSCLC. Normal immortalized epithelial cell line BEAS-2B and four lung cancer cell lines were used for our study. qRT-PCR was used to detect the expression of circ_ZNF124. As indicated, circ_ZNF124 expression was much higher in these lung cancer cell lines than normal epithelial cell BEAS-2B (Fig. 1c, **P < 0.01).

### Knockdown of circ_ZNF124 induces cell cycle arrest and decreases cell growth, colony formation and migration

**Fig. 2:**
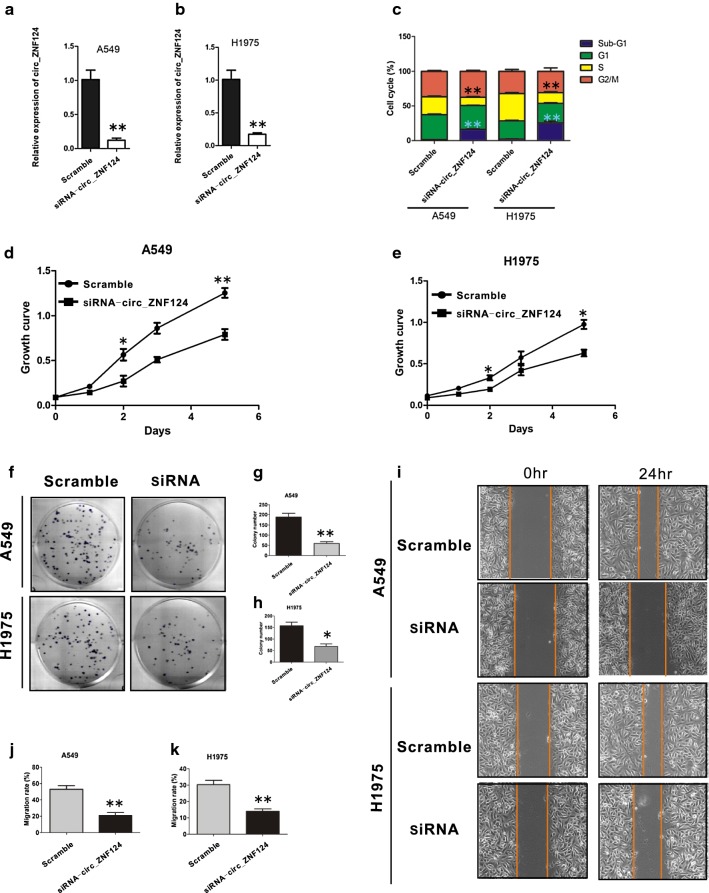
Circ_ZNF124 promoted NSCLC cells proliferation, migration and colony formation. **a**, **b** qRT-PCR results of circ_ZNF124 expression after siRNA knock down in A549 and H1975. **c** Cell cycle detect after knock down of circ_ZNF124. **d**, **e** Cell growth was impaired after interfering circ_ZNF124 expression compared with scramble control. **f** Representative images of cell colony formation. **g**, **h** Statistic results of colony number in scramble and after circ_ZNF124 knock down. **i** Representative images of cell migration with or without circ_ZNF124 knock down. **j**, **k** Statistic results of cell migration. At least 3 replicates were used for analysis. *P < 0.05, **P < 0.01

To further characterize the roles of circ_ZNF124 in NSCLC, A549 and H1975 were randomly selected for downstream study. siRNA that specifically target the junction of the covalently joined 3′ and 5′ ends was used to inhibit circ_ZNF124 expres-sion. siRNA transfection efficiently downregulated circ_ZNF124 expression in A549 and H1975 (Fig. 2a, b, **P < 0.01), A549 and H1975 cells cycle were also arrested in sub-G1 when circ_ZNF124 was knocked down (Fig. 2c, **P < 0.01). In addition, cell growth assay demonstrated that silencing circ_ZNF124 greatly decreased A549 and H1975 growth rate even at the early times after seeding the cells. (Figure 2d, e, **P < 0.05, **P < 0.01). Cell colony formation and migration assay suggested that inhibition of circ_ZNF124 could also impair A549 and H1975 colony formation and cell migration ability (Fig. 2f–k, *P < 0.05, **P < 0.01). These results revealed that circ_ZNF124 plays an important role in the proliferation of NSCLC.

### miR-337-3p is a target of circ_ZNF124 in vivo

**Fig. 3:**
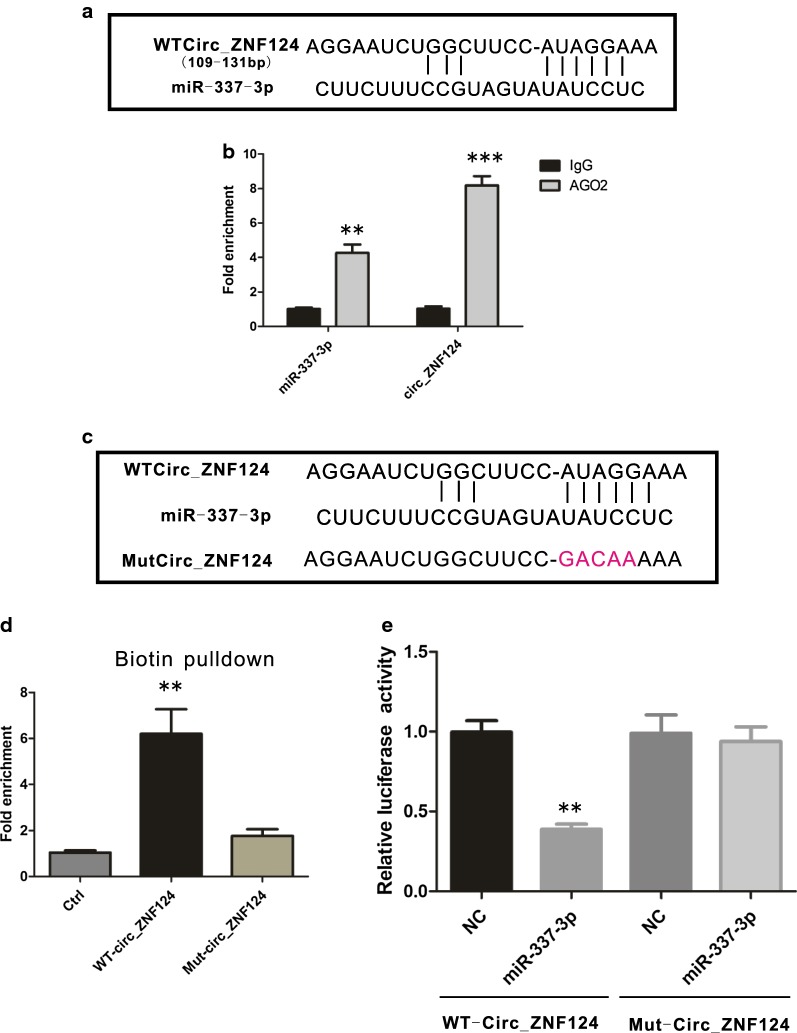
miR-337-3p was the target gene of Circ_ZNF124 in NSCLC. **a** Predicted binding site of miR-337-3p in Circ_ZNF124. **b** qRT-PCR detect miR-337-3p and Circ_ZNF124 expression after RNA pull down with AGO2 antibody in A549 cell line. **c** schematic graph of wild type and mutated miR-337-3p binding sites in Circ_ZNF124. **d** Pull down miR-337-3p with biotin-labeled wild type Circ_ZNF124 or miR-337-3p binding sites mutated Circ_ZNF124 from A549 cell lysates. **e** Reporter assay to detect wild type or miR-337-3p binding sites mutated Circ_ZNF124 activity in A549 cell line. *NC* negative controls.*P < 0.05, **P < 0.01

Studies have shown that one of the important roles of circRNA is to remove the inhibitory effects of miRNA on downstream target genes by adsorbing miRNA [14]. To find which miRNA is the target of circ_ZNF124, we investigated the circinteractome database. Bioinformatics prediction showed that miR-337-3p was scored the highest in all the circ_ZNF124-miRNA matches. The potential binding site of miR-337-3p in circ_ZNF124 as indicated (Fig. 3a). RIP assay with anti-Ago2 antibody showed that both miR-337-3p and circ_ZNF124 can be efficiently pulled down by Ago2 antibody compared with the control IgG (Fig. 3b, **P < 0.01). To test whether circ_ZNF124 can directly interact with miR-337-3p in vivo, we synthesized biotin-labeled WT circ_ZNF124 or miR-337-3p binding site mutated circ_ZNF124 (Fig. 3c) and used them to precipitate miR-337-3p from cell lysates. As expected, WT circ_ZNF124 efficiently pulled down miR-337-3p, while mutated circ_ZNF124 failed to pull down miR-337-3p (Fig. 3d, **P < 0.01). Luciferase assay with WT or miR-337-3p binding sites mutated circ_ZNF124 ligated into PGL3-Luc vector suggested that overexpression of miR-337-3p suppressed the luciferase activity of the circ_ZNF124 WT construct, but not the miR-337-3p binding sites mutated circ_ZNF124 construct in A549 cells (Fig. 3e, **P < 0.01). Thus, these results indicated that miR-337-3p is a target of circ_ZNF124 in vivo.

### miR-337-3p is downregulated in NSCLC cells and is associated with NSCLC cells proliferation

**Fig. 4:**
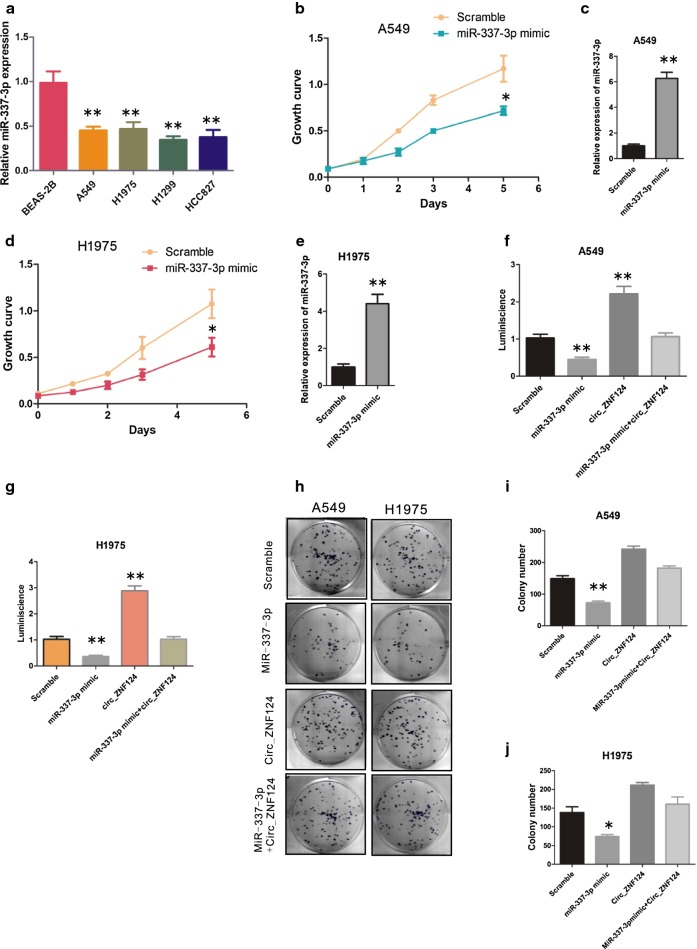
Circ_ZNF124 suppressed miR-337-3p induced NSCLC cells growth arrest. **a** qRT-PCR detect miR-337-3p expression, U6 was used to normalize miR-337-3p expression. miR-337-3p over-expression significantly inhibited **b** A549 and **d** H1975 growth rate. The expression of miR-337-3p in **c** A549 and **e** H1975 were detected by qRT-PCR. Circ_ZNF124 rescued **f** A549 and **g** H1975 cell viability impaired by miR-337-3p. **h** Representative images of cell colony formation. **i**, **j** Statistic results of colony number after cell outgrowth for 14 days. *P < 0.05, **P < 0.01

To determine whether miR-337-3p is associated with NSCLC, qRT-PCR was used to determine the expression level of miR-337-3p in NSCLC cells and normal BEAS-2B cell. As shown in Fig. 4a, miR-337-3p expression level was significantly downregulated in NSCLC cells compared with normal cells (Fig. 4a, **P < 0.01). We next asked whether miR-337-3p plays a role in NSCLC. miR-337-3p mimic was transfected into A549 and H1975, the effect of miR-337-3p on cell growth was evaluated. As indicated, upregulation of miR-337-3p significantly inhibited A549 and H1975 growth compared with scramble control (Fig. 4b–e, *P < 0.05, **P < 0.01). To further test if circ_ZNF124 can sponge miR-337-3p induced cell growth arrest. A549 and H1975 were transfected with miR-337-3p mimic in the presence or absence of circ_ZNF124, cell viability was determined by CellTiter-Glo luminescent on day 5 after transfection. As indicated, cell growth arrest induced by miR-337-3p mimic was greatly reversed by the presence of circ_ZNF124 in A549 and H1975 cell lines (Fig. 4f, g, **P < 0.01). The effect of miR-337-3p on A549 and H1975 cell colony formation was also investigated, as expected, miR-337-3p significantly reduced A549 and H1975 cells colony formation ability, while this effect could also be rescued by circ_ZNF124 (Fig. 4h–j, *P < 0.05, **P < 0.01).

### Identification of JAK2 as the new target gene of miR-337-3p in NSCLC

**Fig. 5:**
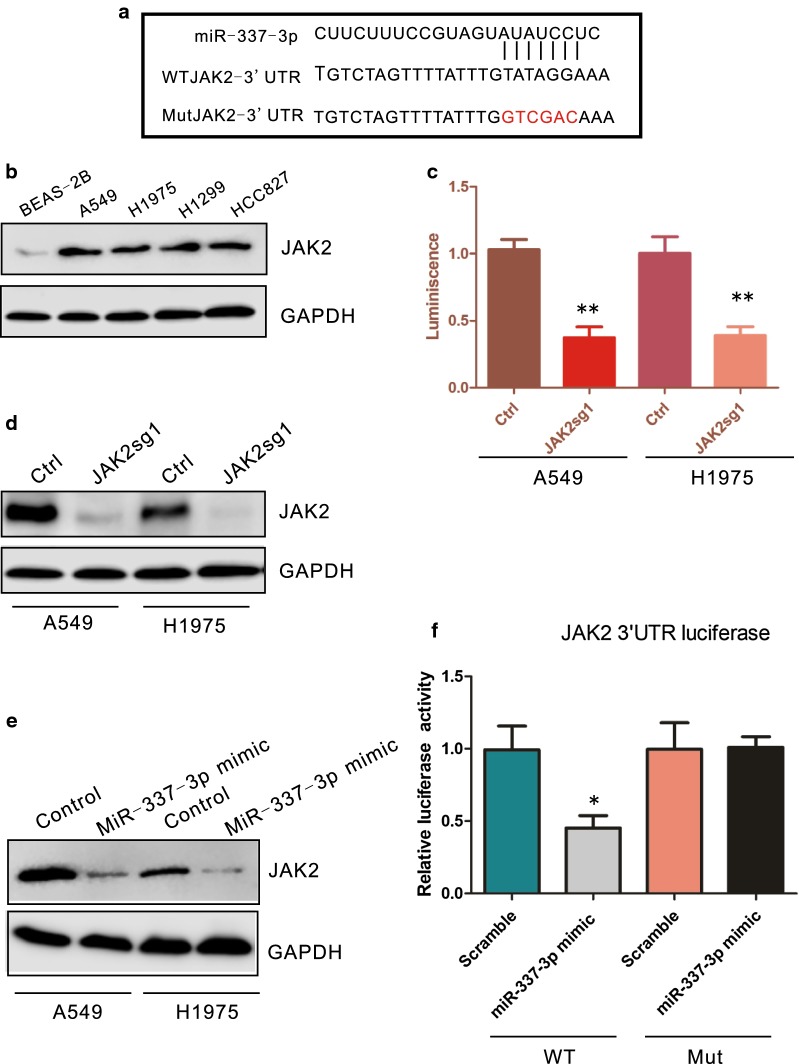
JAK2 is a target of miR-337-3p in NSCLC. **a** Predicated binding site of miR-337-3p in JAK2 3′UTR. **b** Western blots detect JAK2 expression, GAPDH was used as a loading control. **c** CRISPR-cas9 mediated JAK2 depletion impaired A549 and H1975 cells viability. **d** Western blots results of JAK2 deletion. **e** Western blots detect JAK2 expression after over-expressing miR-337-3p in A549 and H1975. **f** Luciferase assay results of wild type (WT) JAK2 3′UTR or presumably miR-337-3p binding site mutated JAK2 3′UTR activity in the presence or absence of miR-337-3p mimic in A549

To further explore the targets of miR-337-3p in NSCLC, TargetScan and miRBase database were used. Analyze results suggested that JAK2 was on the top list of miR-337-3p candidate targets. The potential binding site of miR-337-3p on JAK2 3′-UTR was shown in Fig. 5a. The correlation between JAK2 expression and NSCLC was first investigated. Western blot results showed that JAK2 was significantly higher expressed in lung cancer cells compared with normal epithelial cell (Fig. 5b). Moreover, JAK2 deletion heavily impaired A549 and H1975 cell viability (Fig. 5c, d, **P < 0.01). These results suggested that JAK2 might be involved in NSCLC development. To test if miR-337-3p can directly regulate JAK2 expression in NSCLC. miR-337-3p mimic was transfected into A549 and H1975. Western blot results indicated that miR-337-3p mimic greatly decreased JAK2 protein expression (Fig. 5e). Luciferase assay further demonstrated that miR-337-3p could significantly inhibit WT JAK2 3′UTR luciferase activity with no effect on miR-337-3p binding site mutated JAK2 3′UTR (Fig. 5f, *P < 0.05). These results indicated that JAK2 is important for NSCLC cell growth and it is one of the target gene of miR-337-3p.

### circ_ZNF124 functions as a ceRNA by inhibiting miR-337-3p induced JAK2/STAT3 signaling pathway inactivation

**Fig. 6:**
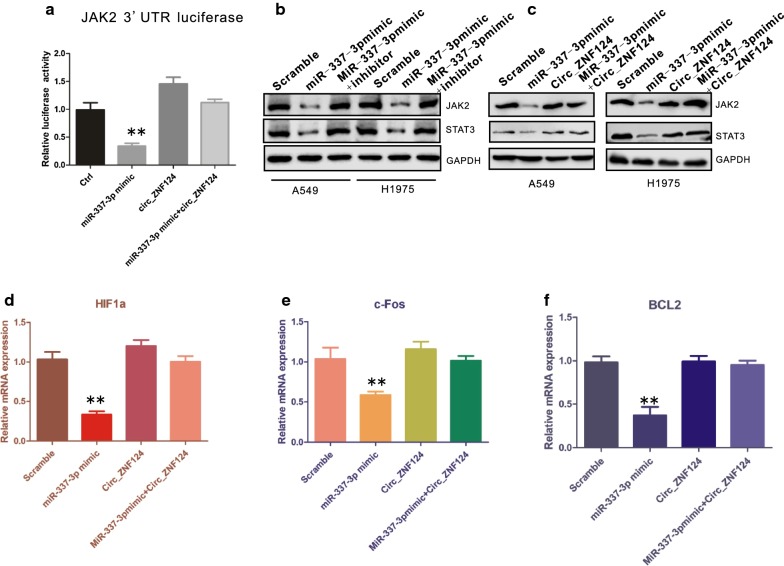
Circ_ZNF124 rescued JAK2/STAT3 signaling pathway activity by acting as miR-337-3p sponge. **a** Luciferase assay results of JAK2 3′UTR activity. **b** miR-337-3p inhibitor rescued JAK2 and STAT3 expression induced by miR-337-3p. **c** Circ_ZNF124 recovered miR-337-3p down-regulated JAK2 and STAT3 protein level. GAPDH was used as loading control. JAK2/STAT3 downstream genes **d** HIF1a, **e** c-Fos and **f** BCL2 expression after over-expression of miR-337-3p mimic in the presence or absence of Circ_ZNF124

Dysregulation of JAK2/STAT3 signaling pathway has been described in many cancers including NSCLC. Previous studies showed that STAT3 was also a target of miR-337-3p. Here, we demonstrated that circ_ZNF124 could inhibit miR-337-3p mediated cell growth arrest and impaired colony formation. we next want to know whether circ_ZNF124 could also rescue miR-337-3p induced downregulation of JAK2/STAT3 signaling pathway. The effect of circ_ZNF124 on rescuing miR-337-3p impaired JAK2 3′UTR activity was first examined. Luciferase assay results showed that miR-337-3p impaired JAK2 3′UTR activity was completely restored in the presence of circ_ZNF124 (Fig. 6a, **P < 0.01). To confirm that JAK2-STAT3 signaling pathway can be directly regulated by miR-337-3p, we co-transfected miR-337-3p with or without miR-337-3p inhibitor, as indicated, the presence of miR-337-3p inhibitor completely abolished miR-337-3p mediated JAK2 and STAT3 down-regulation (Fig. 6b). Furthermore, the down-regulated protein levels of JAK2 and STAT3 caused by miR-337-3p were also rescued by circ_ZNF124 (Fig. 6c). To further test the roles of circ_ZNF124-miR-337-3p in JAK2/STAT3 signaling pathway, JAK2/STAT3 downstream regulated genes BCL2, c-FOS and HIF1a were tested in the presence or absence of circ_ZNF124 and miR-337-3p. As indicated, miR-337-3p induced JAK2/STAT3 signaling pathway downregulation also suppressed its downstream genes BCL2, c-FOS and HIF1a expression. However, the presence of circ_ZNF124 greatly rescued JAK2/STAT3 activity and BCL2, c-FOS and HIF1a expression affected by miR-337-3p (Fig. 6d–f, **P < 0.01). Thus, these results suggested that circ_ZNF124 can function as a sponge of miR-337-3p and rescue miR-337-3p induced inactivation of JAK2/STAT3 signaling pathway in NSCLC.

## Discussion

Despite great advances in the treatment of NSCLC through the use of radiotherapy [4], surgery [26], chemotherapy, and other interventions, the 5 year survival rate of NSCLC patients is still low [36]. The lack of efficient NSCLC biomarkers limited the development of targeted chemical therapy. Nowadays, adjuvant chemotherapy remains the standard of care for patients with resected NSCLC. Among them, cisplatin-based postoperative chemotherapy cisplatin is a widely used chemotherapeutic drug in the clinical treatment of NSCLC [21, 33]. However, due to the genetic background diversity in NSCLC patients, endogenous and acquired drug resistance limits its clinical efficacy [23]. Therefore, identify early NSCLC diagnostic biomarkers and novel molecular targets is of clinical important for NSCLC treatment.

With the advancement of RNA-seq technologies and the development of bioinformatic analysis, more and more circRNAs have been identified [8, 13, 40]. CircRNA is a type of non-polyadenylated circular RNA which is produced during transcription [6]. Recently, more and more research has focused on the functional studies of circular RNA. For example, Ashwal-Fluss showed that some circRNA co-transcription with mRNA from the same locus could function as an RNA trap through competing with their corresponding mRNA expression [1]. Hansen proved that circRNA ciRS-7 can inhibit miRNA miR-7 activity by serving as miR-7 sponge [14]. Furthermore, in contrast to our previous concept, some researchers have demonstrated that like mRNA, some circRNA can also encode proteins, and these circRNA encoded proteins are also functional in cells [19, 29, 38]. Despite molecular mechanism studies, the roles of circRNA in cancers have also been extensively investigated. By using circRNA microarray, Li identified that hsa_circ_0004277 was downregulated in acute myeloid leukemia, and chemotherapy treatment could significantly restore hsa_circ_0004277 expression, indicating that hsa_circ_0004277 could be a new biomarker for Acute Myeloid Leukemia [22]. In the research of bladder carcinoma, Zhong discovered that circTCF25 was highly expressed in bladder carcinoma, further mechanism studies demonstrated that circTCF25 could promote bladder carcinoma cell line proliferation and migration through suppressing miR-103a-3p and miR-107 expression, and increasing CDK6 expression [41].

Previous studies showed that circZNF_124 was upregulated in lung adenocarcinoma, however, the function of circZNF_124 in NSCLC is still unknown. In this study, we provided insights into the clinical significance, function and molecular mechanism of circZNF_124 in NSCLC. The potential association of circZNF_124 with lung cancer cells proliferation was first investigated. The findings indicated that circZNF_124 expression was highly upregulated in NSCLC cells compared with normal epithelial cells. these results indicated that NSCLC could be a potential novel biomarker for NSCLC diagnosis and prognosis.

To interrogate the function of circZNF_124 on NSCLC, siRNA was used to silence circZNF_124 expression. The results showed that abolishing circZNF_124 expression promoted cell cycle arrested in sub-G1 phase and significantly decreased NSCLC cell line A549 and H1975 growth rate, suggesting the oncogenic role of circZNF_124 in NSCLC. Recent studies on circRNA indicated that many circRNA may function as a sponge of miRNA. For example Chi showed that Circular RNA circPIP5K1A promotes NSCLC proliferation and metastasis through targeting miR-600 [9]. Wan found that by acting as a ceRNA, circ_0020123 released miR-488-3p mediated ADAM9 downregulation, which further promoted NSCLC progression [37]. To understand the oncogenic mechanism of circZNF_124 in NSCLC, we used circinteractome to predict potential circZNF_124 targets. Results showed that miR-337-3p was scored the highest among all the potential miRNA targets. RNA immunoprecipitation confirmed their directly interaction in vivo. Luciferase assay showed that miR-337-3p could greatly affect circZNF_124 activity through binding to circZNF_124. These results implicated that miR-337-3p is the target of circZNF_124 in NSCLC.

We next asked whether miR-337-3p is also associated with NSCLC development. The expression and the effect of miR-337-3p on NSCLC were investigated. Contrary to circZNF_124, miR-337-3p expression was downregulated in NSCLC cells compared with normal cell. However, whether circZNF_124 can directly regulate miR-337-3p expression needs further investigation. The roles of miR-337-3p in NSCLC were also investigated. As indicated, miR-337-3p mimic significantly suppressed NSCLC cell lines A549 and H1975 growth rate and colony formation ability. While in the presence of circZNF_124 the function of miR-337-3p in NSCLC is heavily impaired.

Dysregulation of JAK2/STAT3 signaling pathway is associated with many cancer progression and metastasis [5, 20, 39]. miRDB and TargetScan prediction indicated that JAK2 is the target of miR-337-3p. Indeed, when miR-337-3p mimic was transfected into A549 and H1975, JAK2 protein level was greatly reduced, luciferase assay confirmed the regulatory role of miR-337-3p on JAK2 is through directly binding to JAK2 3′UTR, indicating JAK2 is the target of miR-337-3p. Previous studies showed that STAT3 is also a target of miR-337-3p, in consistent with this study, we found that overexpression of miR-337-3p downregulated both JAK2 and STAT3, implicating multi-targets of miR-337-3p. BCL2, c-FOS and HIF1a are transcription factors, whose dysregulation were broadly reported to be associated with tumor inflammation, angiogenesis, and suppression of apoptosis among others [10, 25, 28, 34, 35]. As the downstream genes of JAK2/STAT3 signaling pathway, we found that impaired JAK2/STAT3 signaling pathway mediated by miR-337-3p also downregulated BCL2, c-FOS and HIF1a expression, while circZNF_124 rescued JAK2 activity and these genes expression caused by miR-337-3p. These results strongly support the hypothesis that circZNF_124 facilitates JAK2/STAT3 signaling pathway activation by acting as a competing endogenous RNA (ceRNA) of miR-337-3p.

## Conclusion

Our study provided clinical significance of circZNF_124 in NSCLC and revealed its roles in NSCLC cell proliferation. Mechanically, we demonstrated that circZNF_124 could inhibit miR-337-3p induced JAK2/STAT3 downregulation, promoting NSCLC development. Thus, circZNF_124 could be a potential biomarker or molecular target for NSCLC treatment.

## Data Availability

All data generated or analysed during this study are included in this published article.

## Abbreviations

JAK2: Janus Kinase 2
NSCLC: non-small cell lung cancer
STAT3: signal transducer and activator of transcription 3
HIF1a: hypoxia-inducible factor 1-alpha
BCL2: B-cell lymphoma 2
3′-UTR: 3′-untranslated region
qRT-PCR: quantitative real-time PCR
PI: propidium iodide
SDS-PAGE: sodium dodecyl sulphate–polyacrylamide gel electrophoresis
CTG: CellTiter-Glo
NC: nitrocellulose membrane
RT: room temperature
RIP: RNA immunoprecipitation
GAPDH: glyceraldehyde 3-phosphate dehydrogenase
CRISPR: clustered regularly interspaced short palindromic repeats
gDNA: genomic DNA
